# Color and genesis of californite from Pakistan: insights from μ-XRF mapping, optical spectra and X-ray photoelectron spectroscopy

**DOI:** 10.1038/s41598-019-57186-0

**Published:** 2020-01-14

**Authors:** Zhiyun Lu, Xuemei He, Chenlu Lin, Lin Liang, Xinyu Jin, Qingfeng Guo

**Affiliations:** 1 School of Gemmology, China University of Geosciences, Beijing, 100083 China; 2 School of Earth and Space Sciences, Peking University, Beijing, 100871 China

**Keywords:** Mineralogy, Applied physics

## Abstract

Four californite samples from Pakistan with yellowish-green, green and reddish-brown colors were investigated by combining the methods of μ-XRF mapping, XRD, Raman spectra, optical spectra, EPMA and XPS. The results show that the californite is composed mainly of microcrystalline vesuvianite and smaller amounts of clinochlore. Based on the distribution of the clinochlore, the californite can be divided into three types. The gem-quality californite is composed of microcrystalline vesuvianite and has a translucent appearance. The ordinary-quality californite contains microcrystalline vesuvianite as well as clinochlore, and it has an opaque appearance. The transitional-type has properties that are intermediate between those of gem- and ordinary-quality californite. Octahedrally coordinated iron and chromium in the clinochlore reduce the transparency and contribute to the opaque green and yellowish-green colors of the californite. At sites where there is no clinochlore, Cr^3+^ in the octahedrally coordinated site Y3 of the vesuvianite is mainly responsible for the green tone of the californite, Fe^3+^ and Mn^3+^ at the Y3 site contribute mainly to the yellowish-green and reddish-brown colors, respectively. The Fe^2+^ → Fe^3+^ charge transfer also occurs in vesuvianite and partly influences the appearance of the californite. The actual color of californite that lacks clinochlore is due to the synergy of Cr^3+^, Fe^3+^ and Mn^3+^ crystal field transfers at the octahedral site Y3 as well as the Fe^2+^ → Fe^3+^ charge transfer in the vesuvianite. Vesuvianite in the californite can be assigned to the *P*4/*n* space group, and the occurrence of clinochlore reflects the fact that the californite from Pakistan formed under medium-grade metamorphic conditions at temperatures of ~300–500 °C. The content of clinochlore provides a basis for grading the quality of the californite.

## Introduction

Californite usually refers to a microcrystalline interlocking mineral growth with vesuvianite as the major constituent. Because of its similar appearance to jadeite, californite is often considered as jadeite, and it is commonly known as golden-green jade in Chinese jewelry market. Vesuvianite is an ortho-disilicate mineral with the general formula X_18_X4Y1Y2_4_Y3_8_T_0–5_Si_18_O_68_(O, OH, F)_10_, where the X and X4 sites are occupied by Ca, Na and rare earth elements (REE); the Y1 site by Cu^2+^, Mn^3+^, Fe^2+^ and Al^3+^; and the Y2 and Y3 sites usually by Al^3+^ ^1–8^. As previously reported, elements substituting at the X4, Y1, Y3 and T sites have a great influence on the appearance of vesuvianite, a vesuvianite crystal may appear gold to deep red to opaque brown-black when some of Ca at the X4 site has been substituted by REE, and the intensity of the vesuvianite color increases with increasing REE content^9^. Mn^3+^ and Cu^2+^ can replace Fe^3+^, Mg^2+^, Al^3+^ and Fe^2+^ at the square pyramidal coordination site Y1 and cause the vesuvianite crystal to present yellow or dark red appearance with a lilac hue^5,6,10,11^. Cr^3+^ at the octahedrally coordinated site Y3 is responsible for the green appearance of vesuvianite^12^. In addition, the triangularly and/or tetrahedrally coordinated site T hosts elements like Be and B^13,14^, which accounts for the brown to black and cinnamon-red color of vesuvianite^15–17^.

Vesuvianite is a widespread rock-forming mineral that occurs principally in contact metamorphic zones associated with limestone^18,19^, and the minerals formed in association with the vesuvianite may influence the color of the californite. As previously reported, there is a systematic dependence of the associated minerals on the grade of metamorphism and the crystallization temperature of the vesuvianite, so hydrogarnet (CaAl–CaFe^3+^ series), xonotlite, natrolite–thomsonite, pectolite and calcite are associated minerals at low-grade metamorphisms (<300 °C); grossular, diopside, Mg-chlorite, prehnite, epidote and calcite at medium-grade metamorphisms (~300–500 °C); grossular, diopside, wollastonite, calcite, monticellite, melilite and quartz at higher-grade metamorphic conditions (>500 °C)^1,8,19^.

The major element chemical compositions of minerals are usually assessed by using X-ray fluorescence (XRF), electron probe microanalysis (EPMA) and scanning electron microscope worked with energy dispersive X-ray spectroscopy (SEM–EDS) methods^12,20^. EPMA is applied only on polished sections or special targets. The SEM–EDS system has a faster speed of analysis than EPMA, which makes it possible to obtain element distribution maps within an acceptable time, but the analytical sample is limited to a small size^21^. In contrast, XRF is a nondestructive technique with a relative precision typically approaching 1%, and it usually has a large-size sample stage, and XRF has therefore been applied widely to geological and archaeological samples^22–25^. Recent developments in instrumentation and automation techniques have been applied to XRF, thus producing spectrometers for micro X-ray fluorescence (µ-XRF), and the use of polycapillary X-ray optics guarantees high fluorescence intensities even on the smallest analytical spots of <10 µm^20,26,27^. Up to the present, the color and genesis of vesuvianite have usually been investigated using EPMA, SEM–EDS and optical spectra, and only a few elemental maps have been produced by the µ-XRF mapping of californite.

Accordingly, for this paper we report on our investigation into the color and color genesis of yellowish-green, green and reddish-brown californite using a combination of powder X-ray diffraction (XRD), EPMA, µ-XRF mapping, Raman spectra, optical absorption spectra and X-ray photoelectron spectroscopy (XPS) methods. The aim was to investigate and establish differences in the genesis of multicolored californite and colored vesuvianite single crystals and to explore the factors that affect the quality of californite, thus providing a possible basis for grading its quality.

## Materials and Methods

**Figure 1:**
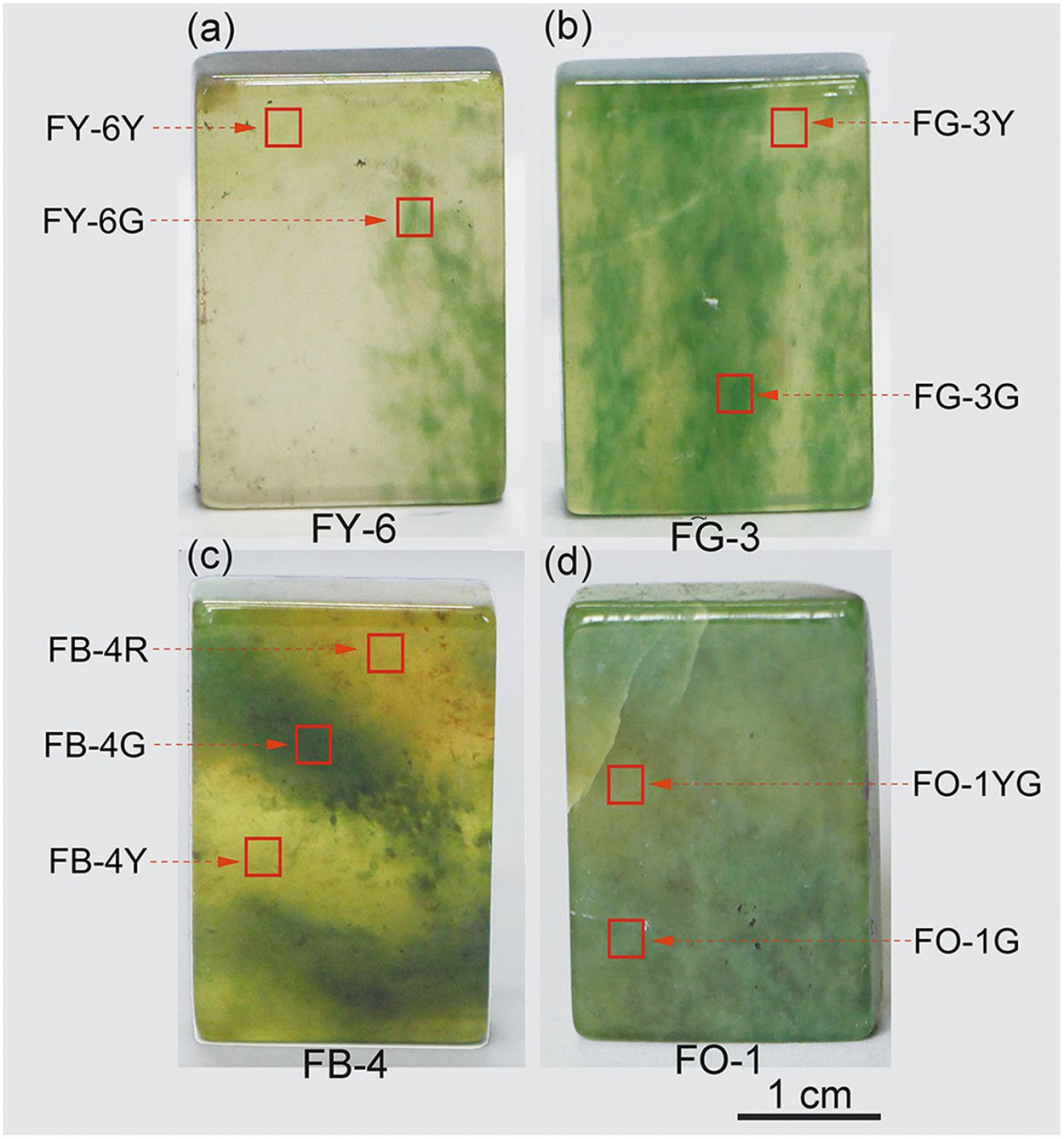
Californite samples from Pakistan with a yellowish-green to green to reddish-brown appearance.

The californite samples came from the border between Pakistan and Afghanistan, four californite samples collected from Pakistan were characterized by color ranging from yellowish-green to green to reddish-brown and labelled FY-6, FG-3, FB-4 and FO-1. The samples were cut and polished into blocks in the size of 3.0 cm × 2.0 cm × 0.6 cm for µ-XRF mapping, optical absorption spectra, Raman spectra and EPMA analysis. These measurements have been carried out on the selected sites of different colors shown in Fig. 1. The scraps were pulverized to 300 mesh powders for powder x ray diffraction (XRD) analysis. Except XRD analysis, other measurements were performed in situ and at room temperature.

The refractive index was measured by a GR-6 gemological refractometer combined with diiodomethane and an accuracy of 0.01, the specific gravity was valued by using an ETNALN ET-320S specific gravity balance with an accuracy of 0.01.

The powder XRD date were acquired with a Rigaku D/Max-RC diffractometer at Institute of Earth Science, China University of Geosciences, Beijing (CUGB). The system was equipped with a conventional copper target x-ray tube set to 45 kV, 200 mA and a graphite monochromator, the scanning speed is 2°/min in the range of 3° < 2θ < 70°. The results were processed by using the Search–Match software package with the International Center of Diffraction Data (ICDD). A more detailed XRD investigation was carried out on the stepping scanning mode at 1 s/step and a step length of 0.02° for collecting X-ray diffraction signals.

The chemical analyses results were verified by traditional EPMA method on a JXA 8230 microprobe at conditions of 15 kV, 20 nA and spot size of 1–10 µm, the signal was collected by using wavelength-dispersive spectrometers. The results were calibrated by natural and synthetic minerals.

The elemental maps of californite samples were measured by using a Bruker Micro-XRF spectrometer (M4 TORNADO) at Bruker Optics Shanghai Office, the spectrometer is equipped with an X-ray tube (Rh anode) with a polycapillary X-ray optic, which can collect the X-rays from the tube and focus them onto smallest sample areas with a diameter <20 µm (Mo Kα). The system worked at 50 kV and 600 µA, a vacuum pump is used to provide a vacuum environment allowing detection to Na. The signal excited by californite was detected by a single 30 mm^2^ SDD enabling count rates of >600 kcps with an acquisition time of 10 ms per pixel. The date was processed by the mineral analyzer software for M4 TORNADO.

The Raman spectra were measured on the polished blocks with a HORIBA HR-Evolution Raman spectrometer at Gem Research Center, the School of Gemology, CUGB. the system equipped with 50 × magnification objectives, a 600-lines-per-millimeter diffraction grating and a Peltier-cooled, Si-based CCD detector was worked with a 532 nm solid stage laser and the laser power (100 mW at the sample surface) was well below the threshold for thermally induced sample degradation. With this system, sample areas as small as 1 µm can be analyzed. The Raman spectra were all recorded at a range of 100 to 1100 cm^−1^ and the collection time was 10 s, the accumulation was 3 scans.

The color features were analyzed by optical absorption spectra using a Shimadzu UV-3000 spectrophotometer at Gem Research Center, the School of Gemology, CUGB. The spectra were recorded in the wavelength region 300 to 800 nm at interval of 0.5 nm. The incident beam was 5 mm in diameter.

The selected sites of different colors in Fig. 1 were cut into 2 mm × 2 mm squares with uniform color, and the XPS spectra were collected using a Thermo Fisher Scientific Escalab 250Xi spectrometer equipped with an AlKα radiation source (photon energy 1486.6 eV). The test size is 650 μm. The data is collected in a vacuum environment of 1 × 10^−9^ mbar and a constant energy of 50 eV. The resolution of the Fe2p is 0.1 eV, and the C1s peak (284.8 eV) is used to calibrate the location of the binding energy. The data was processed by using the CaseXPS software, Shirley background, Gaussian: Lorentz function = 60:40 to fit the XPS data.

## Results

### Gemological properties

The gemological properties of our representative samples are shown in Table S1. The four californite samples are all multicolored, ranging from yellowish-green to green to reddish-brown and with light to dark tones. Samples FY-6 and FG-3 are of gem-quality with a translucent appearance and no internal impurities, FO-1 is of ordinary-quality with an opaque appearance and the dark-green dotted inclusions make the sample less transparent, FB-4 is of transitional-quality and has properties that are intermediate between those of gem- and ordinary-quality californite, The refractive index value is 1.71 and the specific gravity values from 3.32 to 3.35. Except FB-4G, FO-1G and FO-1YG site with opaque appearance, other sites shown in Fig. 1 are all translucent.

### XRD

**Figure 2:**
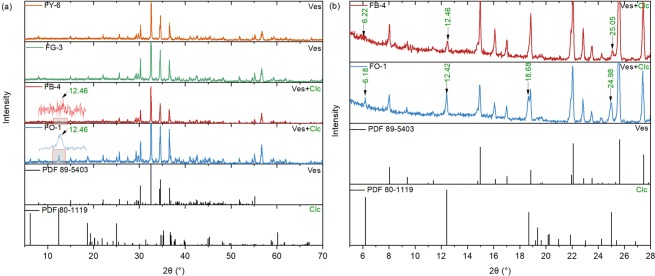
(**a**) Powder XRD patterns of FY-6, FG-3, FB-4 and FO-1 (scanning speed of 2°/min) exhibit the characteristic patterns of vesuvianite (ICDD PDF 89-5403). (**b**) The additional weak diffraction peaks at 2θ = ~12.46° for FB-4 and FO-1 were investigated with a more detailed stepping scanning mode XRD (at a scanning speed of 1 s/step and a step length of 0.02°), which indicated the presence of clinochlore. Ves = vesuvianite; Clc = clinochlore.

Powder XRD data for the californite samples are given in Fig. 2a. The XRD patterns for FY-6, FG-3, FB-4 and FO-1 are all in accordance with the data for vesuvianite (ICDD PDF 89-5403), which indicates the principal mineral in all four californite samples is vesuvianite. There is an additional weak diffraction peak at d = ~7.10 Å (2θ = ~12.46°) for FB-4 and FO-1, so a more detailed XRD investigation of these two samples was carried out on the stepping scanning mode at 1 s/step and a step length of 0.02°, and the results are shown in Fig. 2b. In addition to the diffraction peaks caused by vesuvianite, there are three other peaks at d_(001)_ = 14.20 Å (2θ = 6.22°), d_(002)_ = 7.10 Å (2θ = 12.46°) and d_(004)_ = 3.55 Å (2θ = 25.05°) for FB-4, and four other peaks at d_(001)_ = 14.28 Å (2θ = 6.18°), d_(002)_ = 7.12 Å (2θ = 12.42°), d_(003)_ = 4.74 Å (2θ = 18.68°) and d_(004)_ = 3.56 Å (2θ = 24.98°) for FO-1. All these peaks correspond to the data for clinochlore (ICDD PDF 80-1119), indicating that clinochlore exists in FB-4 and FO-1. The results show that the mineral composition of gem-quality californite is different from that of the transitional- and ordinary-quality californite, and the latter two varieties contain clinochlore.

### EPMA data

The chemical compositions of four californite samples obtained by EPMA are provided in Table 1. The data indicate that in the gem-quality samples FY-6 and FG-3, the yellowish-green and green sites consist of vesuvianite. Clinochlore is present in the ordinary-quality sample FO-1 and the transitional-type sample FB-4, and occurs at the yellowish-green site of FO-1YG1 and the green sites of FB-4G1 and FO-1G1, as confirmed by the crystal-chemical formulae provided in Table 2. However, clinochlore was not found at the yellowish-green and reddish-brown sites of the transitional-type sample FB-4 (FB-4Y and FB-4R).

**Table 1:** Chemical compositions of the californite obtained by EPMA (wt.%).

Oxide	FY-6G	FY-6Y	FG-3G	FG-3Y	FB-4R	FB-4G	FB-4Y	FO-1G	FO-1YG	FB-4G1	FO-1G1	FO-1YG1
SiO_2_	37.61	37.13	37.86	38.43	39.09	38.63	38.49	38.14	37.74	28.10	30.55	30.91
TiO_2_	0.28	0.00	0.00	0.06	0.00	0.17	0.00	0.24	0.00	0.00	0.02	0.04
Al_2_O_3_	18.55	19.46	17.69	18.09	17.44	17.72	17.74	18.01	17.82	26.49	17.60	18.91
Cr_2_O_3_	0.41	0.00	0.09	0.00	0.07	0.17	0.00	0.26	0.07	0.08	0.60	0.05
V_2_O_3_	0.09	0.00	0.00	0.00	0.00	0.00	0.00	0.00	0.00	0.03	0.03	0.03
ZnO	0.00	0.00	0.00	0.00	0.00	0.08	0.00	0.14	0.00	0.08	0.03	0.00
CuO	0.03	0.00	0.00	0.00	0.03	0.00	0.00	0.00	0.00	0.00	0.02	0.05
NiO	0.00	0.03	0.04	0.00	0.02	0.00	0.00	0.05	0.00	0.05	0.05	0.08
CoO	0.00	0.00	0.00	0.00	0.03	0.00	0.00	0.05	0.00	0.02	0.00	0.03
FeO	0.55	0.61	1.13	1.14	0.63	1.02	1.63	2.61	2.57	0.31	7.58	5.25
MnO	0.05	0.11	0.08	0.06	0.25	0.13	0.00	0.16	0.07	0.00	0.08	0.00
MgO	2.81	2.75	3.04	3.00	3.21	3.14	3.01	3.45	3.18	31.45	27.66	29.41
CaO	36.65	36.96	36.99	36.85	37.25	37.16	36.92	35.56	36.04	0.15	0.15	0.01
Na_2_O	0.05	0.00	0.03	0.04	0.05	0.06	0.04	0.00	0.04	0.00	0.01	0.00
K_2_O	0.02	0.00	0.00	0.02	0.02	0.00	0.00	0.00	0.00	0.00	0.00	0.00
P_2_O_5_	0.00	0.04	0.00	0.03	0.00	0.03	0.05	0.00	0.00	0.04	0.01	0.00
SO_3_	0.00	0.00	0.00	0.00	0.00	0.00	0.09	0.00	0.00	0.01	0.00	0.00
F	0.00	0.00	0.00	0.00	0.00	0.00	0.00	0.00	0.00	0.00	0.00	0.00
Cl	0.00	0.00	0.00	0.03	0.00	0.00	0.00	0.00	0.00	0.01	0.00	0.00
Total	97.10	97.09	96.95	97.75	98.09	98.31	97.97	98.67	97.53	86.80	84.39	84.77
Phase	Ves	Ves	Ves	Ves	Ves	Ves	Ves	Ves	Ves	Clc	Clc	Clc

Ves = vesuvianite; Clc = clinochlore.

**Table 2:** Chemical formulae of the clinochlore in californite.

Sites	Chemical formulae
FB-4G1	Mg_4.36_Al_1.51_Fe_0.02_Cr_0.01_Zn_0.01_Ca_0.01_(Si_2.61_Al_1.39_O_10_)(OH)_8_
FO-1G1	Mg_4.10_Al_1.10_Fe_0.63_Cr_0.05_Mn_0.01_Ca_0.02_(Si_3.04_Al_0.96_O_10_)(OH)_8_
FO-1YG1	Mg_4.27_Al_1.18_Fe_0.43_Ni_0.01_(Si_3.01_Al_0.99_O_10_)(OH)_8_

The green color sites FY-6G, FG-3G, FB-4G and FO-1G have higher contents of Cr_2_O_3_ (0.41, 0.09, 0.17 and 0.26 wt.%, respectively) than the yellowish-green color sites FY-6Y, FG-3Y, FB-4Y and FO-1YG (0, 0, 0 and 0.07 wt.%, respectively). The MgO contents in the californite have similar distributions to Cr_2_O_3_, as do TiO_2_, V_2_O_3_ and NiO. Among these elements, only the transitional metals Cr, V and Ni contribute to the green appearance of the californite.

The yellowish-green color sites FY-6Y, FG-3Y and FB-4Y have higher contents of FeO (0.61, 1.14 and 1.63 wt.%, respectively) than the green color sites FY-6G, FG-3G and FB-4G (0.55, 1.13 and 1.02 wt.%, respectively). It is also worth noting that the FeO content in opaque sample FO-1 (4.50 wt.% average) is much higher than in the other samples (0.58, 1.14 and 0.90 wt.% averages). This indicates that the clinochlore exists in an iron-rich environment. The site FB-4R, with its reddish-brown appearance, has a much high content of MnO (0.25 wt.%) than the other sites.

### Elemental mapping by μ-XRF

**Figure 3:**
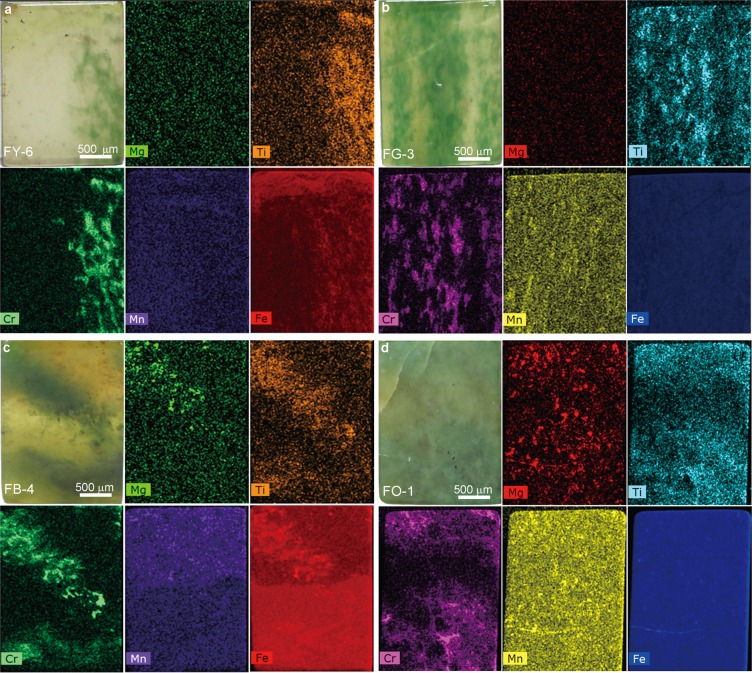
Elemental maps produced by μ-XRF for FY-6 (**a**), FG-3 (**b**), FB-4 (**c**) and FO-1 (**d**). The distributions of Fe, Cr and Mn correspond to the yellowish-green, green and reddish-brown color sites, respectively. The distribution of Mg is linked to the presence of clinochlore.

The elemental maps of californite made by μ-XRF are shown in Fig. 3. The distributions of chromium and titanium correspond well to the green tone distributions in four californite samples, and they have the same distributions in samples of gem-, transitional- and ordinary-quality californite.

In contrast, the magnesium, iron and manganese distributions differ for the three types of californite. In the case of magnesium, it has a uniform distribution in gem-quality samples FY-6 and FG-3, but it is more concentrated at the green sites of the transitional- and ordinary-quality samples, which may be due to the presence of the clinochlore found in FB-4 and FO-1. Iron is distributed in the upper yellowish-green area of FY-6 and the lower yellowish-green area of FB-4. It is also more concentrated at the green sites of FY-6 and FB-4, but shows a homogeneous distribution in FG-3 and FO-1. The distribution of manganese in FB-4 is different from that of iron, and it is concentrated in the reddish-brown area. Manganese shows a uniform distribution in all other samples.

### Raman spectra

**Figure 4:**
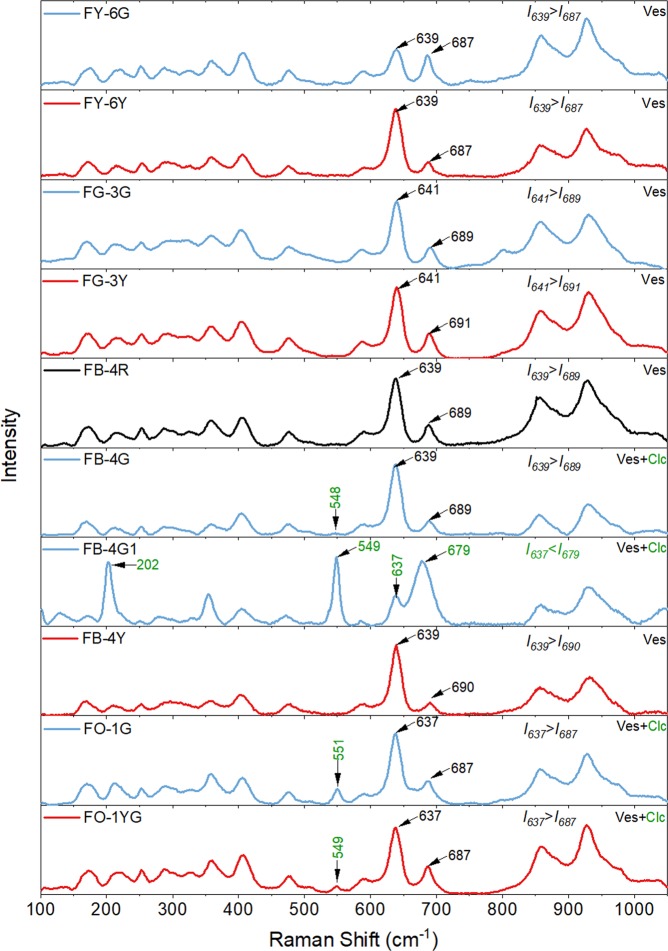
Raman spectra for representative sites of californite samples FY-6, FG-3, FB-4 and FO-1. The red, blue and black lines respectively represent the yellowish-green, green and reddish-brown sites of the californite. Ves = vesuvianite; Clc = clinochlore.

The Raman spectra for the different color sites shown in Fig. 1 are given in Fig. 4. These vibrational peaks or bands are similar to the previously published results for vesuvianite crystals^28,29^, indicating the presence of vesuvianite at all sites. Furthermore, there are two additional bands at 203 and 549 cm^−1^ for the green site FB-4G1, and the band at 551 cm^−1^ also appears on the yellowish-green and green sites of FO-1, and these bands fit the standard data for clinochlore (RRUFF R060725) as well as the Raman spectra for clinochlore measured by Gopal *et al*.^30^. The band at 549–551 cm^−1^ could presumably be attributed to the Si–O_b_–Si stretching mode, and the vibrations at 203 cm^−1^ have been assigned to the brucite Me–OH libration^31^. Remarkably, for FB-4G1 the Raman band at 637 cm^−1^ has a much lower intensity (*I*_637_) than that at 679 cm^−1^ (*I*_679_), while the intensity of the band at 637–639 cm^−1^ is higher than that at 687–691 cm^−1^ in other sites. A new Raman spectrum named FB-4G was collected alongside the position of FB-4G1, for which the intensity *I*_639_ is higher than *I*_689_, and the peak at 203 cm^−1^ is absent. The Raman characteristics of FB-4G correspond to the normal results for the FY-6Y site, so the strong band at 679 cm^−1^ for the FB-4G1 site can be ascribed to the high content of clinochlore in the californite and the peak is assigned to the Si–O–Si symmetric stretching mode of the clinochlore.

The Raman spectra for the yellowish-green and green sites in gem- and ordinary-quality californite show different patterns, and this is because the gem-quality californite contains only vesuvianite while the ordinary-quality californite contains both vesuvianite and clinochlore. The californite sample FB-4 shows transitional-type characteristics, so that the yellowish-green and reddish-brown sites contain only vesuvianite whereas the green site contains both vesuvianite and clinochlore.

As previously reported^1,28,32^, there is a correlation between the crystal habit of vesuvianite and the relative intensities of Raman bands at 637–639 and 687–691 cm^−1^. For vesuvianite that formed at a high temperature, *I*_*637–639*_ > *I*_*687–691*_, and the vesuvianite can be assigned to the *P*4/*nnc* space group, but for vesuvianite formed at a lower temperature, *I*_*637–639*_ < *I*_*687–691*_ and the crystal can be assigned to the *P*4/*n* space group. Furthermore, there is a systematic relationship between space-group symmetry and rod arrangements and the crystallization temperature of vesuvianite, so that a *P*4*nc*-dominant space group represents a temperature of < 300 °C, a *P*4/*n*-dominant space group at ~300–500 °C, and a *P*4/*nnc*-dominant space group at >500 °C. All our samples of californite have Raman spectra with the characteristics *I*_*637–639*_ < *I*_*687–691*_, and XRD analyses show a match with *P*4/*n* space group vesuvianite (ICDD PDF 89–5403). We infer, therefore, that the microcrystalline vesuvianite in our specimens of californite is low vesuvianite that formed in the temperature range of ~300–500 °C^3,18,19,32^.

### Optical spectra

**Figure 5:**
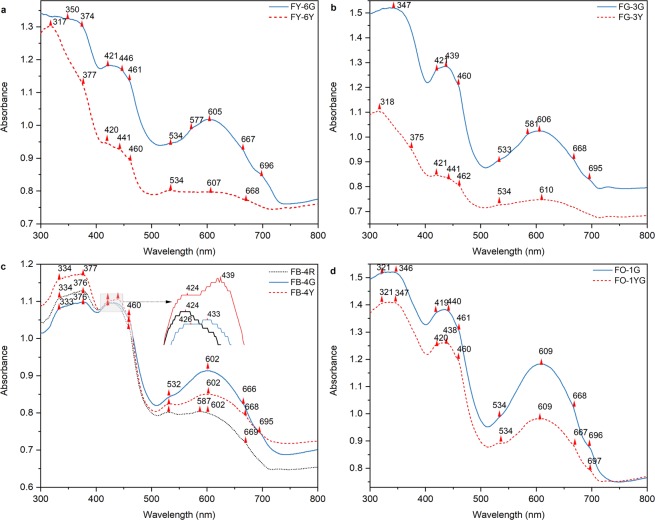
Optical absorption spectra for different color sites on FY-6 (**a**), FG-3 (**b**), FB-4 (**c**) and FO-1 (**d**). The red dashed line, blue solid line and black dotted line respectively represent the yellowish-green, green and reddish-brown zones of the californite samples.

Typical UV-visible absorption spectra for our specimens of californite are illustrated in Fig. 5. Common broad bands occur at 419–426, 438–446 and 602–610 nm and shoulder peaks at 460–462, 532–534 and 666–669 nm. Other bands are also present at 346–350, 374–377, 433, 577–581 and 695–697 nm.

In the UV-visible absorption spectra, the strong broad band observed at 346–350 nm was found at the green sites of gem-quality californite and at the yellowish-green and green sites of ordinary-quality californite. According to the EPMA data, chromium and iron exist together in FY-6G, FG-3G, FO-1YG and FO-1G and the band can therefore be assigned to the synergy of the ^4^A_2g_(F) → ^2^T_1g_(F) transition of Cr^3+^ in octahedral sites and the ^6^A_1g_(S) → ^4^E_g_(D) transition of Fe^3+^ ions^30,33,34^.

The absorption edge near 400 nm found in the UV-visible absorption spectra of all our californite samples is due to octahedral Fe^3+^ in the Al^3+^ site, and it is generally understood to arise from the oxygen-to-metal electronic charge transfer transition O^2−^ → Fe^2+^ or O^2−^ → Fe^3+^ ^35^^,^^36^. The weak shoulder bands at 374–377, 460–462 and 577–581 nm are respectively linked to ^6^A_1g_ → ^4^E_1_(G), ^6^A_1_ → ^4^A_1_E(G) and ^6^A_1g_(S) → ^4^T_1g_(G) transitions due to Fe^3+^ at the octahedral site^15,37–39^.

The strong absorption band centered at 419–426 nm for all sites has been assigned to the ^4^A_2g_(F) → ^4^T_1g_(F) transition caused by Cr^3+^ in the octahedral site^32–34,39,40^. The additional weaker peaks at 433, 438–446, 666–669 and 695–697 nm also indicate the presence of Cr^3+^ ^35^^,^^39^^,^^40^, and the ^5^B_1_(^5^E) → ^5^A_2_(^5^T_2_) transition of Mn^3+^ at the octahedral crystal site can also be present at 440 nm^41^. Accordingly, the band at 438–446 nm is recognized as due to the simultaneous presence of Cr^3+^ and Mn^3+^ at the octahedral crystal site. The band at 532–534 nm has been assigned to the ^5^B_1_(^5^E) → ^5^A_1_(^5^T_2_) transition of Mn^3+^ at the octahedral site^41^.

As previously reported, the ^4^A_2g_(F) → ^4^T_2g_(F) transition caused by Cr^3+^ in the octahedral site is represented by a strong absorption band centered at about 600 nm^33–35,42,43^, and the Fe^2+^ → Fe^3+^ charge transfer is also represented by a broad absorption band at around 600 nm in ruby^44^. Accordingly, in the absorption spectra for our specimens of californite, we ascribe the band centered at 600 nm to the simultaneous presence of the ^4^A_2g_(F) → ^4^T_2g_(F) transition caused by Cr^3+^ and the Fe^2+^ → Fe^3+^ charge transfer, Cr^3+^ plays a dominant role at the green site, while the Fe^2+^ → Fe^3+^ charge transfer plays a dominant role at the yellowish-green site.

For the yellowish-green sites, the strong absorption occurs only at 420–421, 438–441 and 460–462 nm and the absorption intensity is weak in the range of 500–700 nm. The green sites at 532–534, 602–610 and 666–669 nm have a higher band or peak intensity than the yellowish-green sites, causing an absorption minimum at about 510 nm and producing the green tone. The reddish-brown and yellowish-green sites present similar spectra, but the absorption at 532 nm of the reddish-brown site of FB-4R is stronger than that at the yellowish-green sites, resulting a strong absorption from 400 to 550 nm and producing the reddish-brown tone.

### XPS

**Figure 6:**
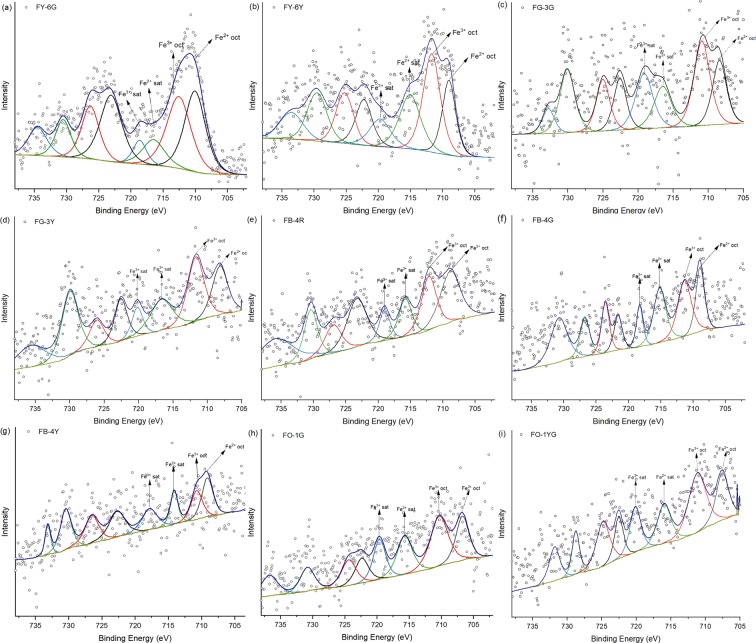
Shirley background-subtracted XPS spectra in the Fe region of green sites (**a**,**c**,**f**,**h**), yellowish-green sites (**b**,**d**,**g**,**i**) and reddish-brown site (**e**). The black, red, green and blue peaks represent octahedral Fe^2+^, octahedral Fe^3+^, satellite Fe^2+^ and satellite Fe^3+^, respectively.

The Fe 2p_3*/*2_ spectra of our specimens of californite were fitted using Gaussian–Lorentzian (GL) = 60:40 and additional broad satellite and surface features. The results of XPS and peak fitting are given in Fig. 6 and Table S2. In the region of Fe 2p_3/2_, the Fe^3+^ binding energy values range from 710.2 to 712.3 eV in octahedral coordination and from 712.9 to 713.7 eV in tetrahedral coordination, while the Fe^2+^ binding energy values range from 709.2 to 709.6 eV in octahedral coordination^45,46^. The deconvolution of the Fe 2p_3/2_ spectrum in our work produced two components (706.7–710.0 and 710.3–712.6 eV), which accords well with the results of previous studies^45,46^, so the binding energies at 706.7–710.0 and 710.3–712.6 eV are caused by the octahedral coordination of Fe^2+^ and Fe^3+^, respectively. The peaks at 714.1–716.7 and 717.8–720.2 eV are attributed respectively to the satellite peaks of Fe^2+^ and Fe^3+^.

The XPS spectra characteristics of gem-, transitional- and ordinary-quality californite are basically the same, and consistent with the results of Panikorovskii (2017)^7^, and this indicates that at sites where there is no clinochlore, Fe^3+^ and Fe^2+^ exist in the octahedral coordination site Y3. At the sites of FB-4G, FO-1G and FO-1 where clinochlore exists, Fe^2+^ and Fe^3+^ occupy the octahedral coordination sites of both vesuvianite and clinochlore.

## Discussion

Our systematic investigation of the characteristics of four multicolored californite samples from Pakistan using EPMA, μ-XRF mapping, optical absorption spectra and XPS has demonstrated that the color of californite is closely connected with transition metal elements such as chromium, iron and manganese.

### Chromium in vesuvianite and clinochlore

The EPMA and μ-XRF mapping results show that chromium is concentrated at the green sites in our californite samples, and the XRD results and Raman spectra for the green sites in gem-quality californite are all in accordance with vesuvianite that belongs to the *P*4/*n* space group. In addition, the results show that clinochlore is present at the green and yellowish-green sites of ordinary-quality californite and at the green site of transitional-type californite, which indicates that chromium ions exist in the microcrystalline vesuvianite and clinochlore. In the UV-visible absorption spectra, in agreement with previous studies^33–35,42,43^, the bands centered at 346–350, 419–426 and 602–610 nm in all samples indicate transitions of ^4^A_2g_(F) → ^2^T_1g_(F), ^4^A_2g_(F) → ^4^T_1g_(F) and ^4^A_2g_(F) → ^4^T_2g_(F) caused by Cr^3+^ in the octahedral sites, respectively. Moreover, the weaker peaks at 438–446 and 666–669 nm are also caused by the presence of Cr^3+^ ^39^, and all these absorption bands and peaks produce an absorption minimum at about 510 nm, thus producing the green tone.

Vesuvianite single crystals with a green tone were studied by Bradshaw (1972)^47^, Groat & Hawthorne (1992)^13^ and Kobayashi & Kaneda (2010)^12^, and they showed that the content of Cr_2_O_3_ in vesuvianite may be as high as 6.21 wt.%, and that the chromium probably occurs at the octahedral coordination site Y3, thus giving the chromium-bearing vesuvianite single crystals their usual vivid green color. In previous studies of clinochlore with a green appearance, Cr^3+^ was found to occupy the octahedral sites^48,49^. In our specimens of californite, Cr^3+^ was found in clinochlore at the FB-4G1, FO-1YG1 and FO-1G1 sites, and for the gem-quality californite we found that Cr^3+^ at the octahedral coordination site in vesuvianite affected the green and yellowish-green tones. For ordinary-quality californite, Cr^3+^ at the octahedral sites in both the vesuvianite and clinochlore affected both the opaque green and yellowish-green colors. In the transitional-type californite where there is no clinochlore, Cr^3+^ occurs in the vesuvianite at the octahedral site, but when vesuvianite and clinochlore exist together, the Cr^3+^ occurs in the octahedral sites of both the vesuvianite and clinochlore.

### Iron in vesuvianite and clinochlore

Although not a constituent element of vesuvianite or clinochlore, iron is nevertheless widely found in both minerals. Iron can occupy the square pyramidal coordination site Y1 and octahedrally coordinated site Y3 as Fe^2+^ and Fe^3+^ in vesuvianite^7,13,32^. In the structure of clinochlore, iron can occupy the octahedrally coordinated sites M1 or M2 as Fe^2+^ and M4 as Fe^3+^ ^50^. The distinction between Fe^2+^ and Fe^3+^ is usually made from XPS spectra or UV-visible absorption spectra. The XPS spectra of our specimens of californite in the Fe 2p_3/2_ region exhibit characteristic peaks at 706.7–710.0 and 710.3–712.6 eV, which are caused by the octahedral coordination of Fe^2+^ and Fe^3+^, and there are no obvious differences between the gem-, transitional- and ordinary-quality californites. The absorption edge near 400 nm found in the UV-visible absorption spectra of all our californite samples is due to octahedral Fe^3+^ in the Al^3+^ site, and it is generally understood to arise from the oxygen-to-metal electronic charge transfer transition O^2−^ → Fe^2+^ or O^2−^ → Fe^3+^ ^35,36^. The weak bands at 374–377, 460–462 and 577–581 nm in all our californite samples are linked to ^6^A_1g_ → ^4^E_1_(G), ^6^A_1_ → ^4^A_1_E(G) and ^6^A_1g_(S) → ^4^T_1g_(G) transitions of Fe^3+^, respectively, at the octahedral site^15,37–39^. The Fe^2+^ → Fe^3+^ charge transfer is also represented by a broad band at around 600 nm^44^. Accordingly, we conclude that iron exists in the californite as Fe^3+^ at the octahedral coordination site and that the Fe^2+^ → Fe^3+^ charge transfer is also represented in the spectra, but there is a complex distribution of the spectra among the gem-, transitional- and ordinary-quality californite.

In the gem-quality californite, our analyses have shown that iron exists in the octahedrally coordinated sites Y2 or Y3 as Fe^2+^ and Fe^3+^. It has previously been reported that >90% of the total iron in green vesuvianite crystals with a low titanium content (<1 wt.%) is octahedrally coordinated Fe^3+^ at the Y3 site^51^. The iron in our specimens of gem-quality californite is concentrated at the yellowish-green sites according to the µ-XRF mapping. Moreover, the iron content is higher at the yellowish-green site than at the green site. The Fe^2+^ → Fe^3+^ charge transfer is represented by a broad band at around 600 nm. We conclude, therefore, that the yellowish-green appearance of gem-quality californite is due mainly to the synergy of the Fe^3+^ at the octahedrally coordinated site Y3 and the Fe^2+^ → Fe^3+^ charge transfer of the vesuvianite.

The XRD, Raman spectra and EPMA analyses indicate that iron-bearing clinochlore is widely distributed at the yellowish-green and green sites of ordinary-quality sample FO-1, and is also present in smaller quantities at the green site of the transitional-type sample FB-4. The iron contents in our specimens decrease gradually from the opaque ordinary-quality californite to the transitional- and gem-quality californites. According to Gopal *et al*.^30^ and Liang *et al*.^52^, iron has a significant influence on the green color of clinochlore, and our µ-XRF mapping results for the transitional-type sample FB-4 and the ordinary-quality sample FO-1 show that iron exists where there is magnesium. These data indicate a link between magnesium and the clinochlore. Our data show, therefore, that iron in clinochlore can also contribute to the green color of the transitional- and ordinary-quality californite specimens. According to the mineral assemblages for various metamorphic grades and the crystallization temperatures of vesuvianite^1,3,18,19^, The occurrence of clinochlore reflects that californite was formed during medium-grade metamorphism and at temperatures of ~300–500 °C.

### Manganese in vesuvianite

In a similar way to iron, manganese is widely found in vesuvianite. Manganese can occupy the square pyramidal coordination site Y1 and the octahedrally coordinated site Y3^5,7,8,12^. Manganese in clinochlore usually exists as Mn^2+^ ^30^, and in our specimens the bands in the UV-visible absorption spectra at 438–441 and 532–534 nm indicate the presence of Mn^3+^ and are associated with the ^5^B_1_(^5^E) → ^5^A_2_(^5^T_2_) and ^5^B_1_(^5^E) → ^5^A_1_(^5^T_2_) transitions of Mn^3+^ at the octahedrally coordinated site. Mn^3+^ is presumed to exist in vesuvianite at the octahedrally coordinated site Y3. Similar to the distribution of Fe^3+^, Mn^3+^ is widely distributed in all samples of vesuvianite, but in our samples it is responsible mainly for the reddish-brown color of FB-4 and partly responsible for the appearance of the yellowish-green and green californites. When the MnO content in vesuvianite reaches about 0.25 wt.% (Mn^3+^ 0.34 wt.%), the synergy of the Mn^3+^, Fe^3+^ and Cr^3+^ crystal field transfers at the octahedral site Y3 and the Fe^2+^ → Fe^3+^ charge transfer produces a significant reddish-brown tone.

## Conclusions

Our systematic investigation of four multicolored californite samples from Pakistan demonstrates that the causes of the yellowish-green, green and reddish-brown colors are complex. The californite is composed mainly of microcrystalline vesuvianite and minor amounts of clinochlore. Based on the distribution of the clinochlore, the multicolored californite can be classified into three types. The gem-quality type is translucent and composed of microcrystalline vesuvianite, the ordinary-quality type has an opaque appearance and contains microcrystalline vesuvianite as well as clinochlore, and the third type is transitional between the gem- and ordinary-quality types.

Clinochlore is distributed widely in the yellowish-green and green sites of the ordinary-quality californite, but in the transitional-type californite it is distributed only at the opaque green sites. Furthermore, clinochlore does not occur in any of the translucent gem-quality samples, and it seems, therefore, that the transparency of californite is controlled mainly by the content of clinochlore. Octahedrally coordinated iron and chromium in clinochlore decrease the transparency of the californite, and they contribute in part to the green and yellowish-green colors of the californite. The content of clinochlore can therefore provide a basis for grading the quality of californite. Moreover, the occurrence of clinochlore together with vesuvianite that belongs to the *P*4/*n* space group indicates that the californite from Pakistan formed at temperatures of ~300–500 °C during medium-grade metamorphism^3,18,19^.

At sites where there is no clinochlore, the green tone of the californite is caused mainly by Cr^3+^ at the octahedrally coordinated site Y3 of the vesuvianite, and it is affected to some extent by the weak absorption of Fe^3+^ and Mn^3+^ in the vesuvianite. In addition, the Fe^2+^ → Fe^3+^ charge transfer may also exist in the vesuvianite and contribute to the green tone of the californite. The yellowish-green tone is caused mainly by the Fe^3+^ crystal field transfer at the octahedral coordination site Y3 in the vesuvianite as well as the Fe^2+^ → Fe^3+^ charge transfer. The trace amounts of Cr^3+^ and Mn^3+^ in the vesuvianite also have some effect on the yellowish-green tone. The reddish-brown color of the californite is related mainly to high Mn^3+^ contents in the microcrystalline vesuvianite and it is also partly affected by the presence of Cr^3+^ and Fe^3+^ in the octahedrally coordinated site Y3. The color of californite that lacks any clinochlore is the synergy of the Cr^3+^, Fe^3+^ and Mn^3+^ crystal field transfer at the octahedral site Y3 of the vesuvianite as well as the Fe^2+^ → Fe^3+^ charge transfer.

## Supplementary information

Gemological properties of californite samples and deconvolution parameters in Fe 2p region of californite.

## Data Availability

The dataset for this study is available from the corresponding author upon reasonable request.
