# Supplementary material for: Color and genesis of californite from Pakistan: insights from μ-XRF mapping, optical spectra and X-ray photoelectron spectroscopy
Source: Sci Rep. 2020 Jan 14;10:285. doi: 10.1038/s41598-019-57186-0 (PMC6959299; doi:10.1038/s41598-019-57186-0)
Supplement: Supplementary file 1 — Gemological properties of californite samples and deconvolution parameters in Fe 2p region of californite. [file 41598_2019_57186_MOESM1_ESM.pdf]

Color and genesis of californite from Pakistan: insights from μ-XRF mapping, optical spectra and X-ray photoelectron spectroscopy

Zhiyun Lu[1], Xuemei He[1]*, Chenlu Lin[1], Lin Liang[2], Xinyu Jin[1], Qingfeng Guo[1]

Zhiyun Lu zylu@cugb.edu.cn 1689715061@qq.com Tel:86-17610889299

School of Gemmology, China University of Geosciences, Beijing 100083, China
Address: China University of Geosciences, Beijing No. 29 Xueyuan Road, Haidian District, Beijing, P.R.China 100083
16-digit ORCID: https://orcid.org/0000-0002-0590-2412

Xuemei He Hexuemei3127@126.com Tel:86-13693203127
School of Gemmology, China University of Geosciences, Beijing 100083, China
Address: China University of Geosciences, Beijing No. 29 Xueyuan Road, Haidian District, Beijing, P.R.China 100083
16-digit ORCID: https://orcid.org/0000-0002-5758-4057

Chenlu Lin 542955567@qq.com Tel:86-18813184355
School of Gemmology, China University of Geosciences, Beijing 100083, China
Address: China University of Geosciences, Beijing No. 29 Xueyuan Road, Haidian District, Beijing, P.R.China 100083
16-digit ORCID: https://orcid.org/0000-0002-8754-6078

Lin Liang lin.liang@pku.edu.cn Tel:86-18811312831
School of Earth and Space Sciences, Peking University, Beijing 100871, China
Address: No.5 Yiheyuan Road Haidian District, Beijing, P.R.China 100871

Xinyu Jin 619310297@qq.com Tel:86-15652688134
School of Gemmology, China University of Geosciences, Beijing 100083, China
Address: China University of Geosciences, Beijing No. 29 Xueyuan Road, Haidian District, Beijing, P.R.China 100083

Qingfeng Guo qfguo@cugb.edu.cn Tel: 86-15811141654
School of Gemmology, China University of Geosciences, Beijing 100083, China
Address: China University of Geosciences, Beijing No. 29 Xueyuan Road, Haidian District, Beijing, P.R.China 100083

Additional Information

Table S1. Gemological properties of californite samples

Sample	Sites	Color	Transparency	Refractive index	Specific gravity
FY-6	FY-6G	Green	Translucent	1.71(0)	3.35(3)
	FY-6Y	Yellowish-green	Translucent		
FG-3	FG-3G	Green	Translucent	1.71(0)	3.34(2)
	FG-3Y	Yellowish-green	Translucent		
FB-4	FB-4R	Reddish-brown	Translucent	1.71(0)	3.33(7)
	FB-4G	Green	Opaque		
	FB-4Y	Yellowish-green	Translucent		
FO-1	FO-1G	Green	Opaque	1.71(3)	3.32(0)
	FO-1YG	Yellowish-green	Opaque		

Table S2. Deconvolution parameters in Fe 2p region of californite

Name[a]	Position (eV)								
	FY-6G	FY-6Y	FG-3G	FG-3Y	FB-4R	FB-4G	FB-4Y	FO-1G	FO-1YG
Fe^{2+} oct $2p_{3/2}$	710.0	709.0	708.4	708.2	708.9	708.9	709.1	706.7	707.6
Fe^{3+} oct $2p_{3/2}$	712.6	711.7	710.9	711.7	712.2	711.3	710.8	710.3	711.2
Fe^{2+} sat $2p_{3/2}$	716.5	715.1	716.5	716.7	715.8	715.2	714.1	715.7	716.0
Fe^{3+} sat $2p_{3/2}$	718.6	719.7	719.1	720.2	719.0	718.3	717.8	719.6	720.2
Fe^{2+} oct $2p_{1/2}$	723.1	722.3	722.6	722.6	723.3	721.6	722.7	722.4	722.5
Fe^{3+} oct $2p_{1/2}$	726.3	725.3	725.0	726.1	726.9	723.6	726.5	724.4	724.8
Fe^{2+} sat $2p_{1/2}$	730.5	729.6	730.1	730.0	730.4	726.7	730.5	730.8	728.8
Fe^{3+} sat $2p_{1/2}$	734.5	733.6	732.8	735.6	735.9	730.8	733.1	736.8	731.8

Name[a]	FHWM								
	FY-6G	FY-6Y	FG-3G	FG-3Y	FB-4R	FB-4G	FB-4Y	FO-1G	FO-1YG
Fe^{2+} oct $2p_{3/2}$	3.8	2.5	2.3	2.7	3.6	1.6	2.0	2.4	2.1
Fe^{3+} oct $2p_{3/2}$	4.4	3.2	2.7	2.8	2.7	2.4	2.0	3.2	3.0
Fe^{2+} sat $2p_{3/2}$	4.1	3.8	3.0	3.8	2.4	2.2	1.4	2.8	2.3
Fe^{3+} sat $2p_{3/2}$	2.5	3.8	2.9	1.8	1.9	1.2	2.7	2.3	1.9
Fe^{2+} oct $2p_{1/2}$	3.9	3.0	2.1	2.2	3.6	1.5	2.9	2.5	1.5
Fe^{3+} oct $2p_{1/2}$	3.2	3.2	2.2	2.5	3.0	1.4	2.5	2.7	2.3
Fe^{2+} sat $2p_{1/2}$	2.8	3.6	2.5	2.8	2.5	1.7	2.0	2.6	1.2
Fe^{3+} sat $2p_{1/2}$	3.4	4.3	2.0	5.0	6.7	3.0	1.0	3.1	1.8

Name[a]	% of area								
	FY-6G	FY-6Y	FG-3G	FG-3Y	FB-4R	FB-4G	FB-4Y	FO-1G	FO-1YG
Fe^{2+} oct $2p_{3/2}$	3.8	2.5	2.3	2.7	3.6	1.6	2.0	2.4	2.1
Fe^{3+} oct $2p_{3/2}$	4.4	3.2	2.7	2.8	2.7	2.4	2.0	3.2	3.0
Fe^{2+} sat $2p_{3/2}$	4.1	3.8	3.0	3.8	2.4	2.2	1.4	2.8	2.3
Fe^{3+} sat $2p_{3/2}$	2.5	3.8	2.9	1.8	1.9	1.2	2.7	2.3	1.9
Fe^{2+} oct $2p_{1/2}$	3.9	3.0	2.1	2.2	3.6	1.5	2.9	2.5	1.5
Fe^{3+} oct $2p_{1/2}$	3.2	3.2	2.2	2.5	3.0	1.4	2.5	2.7	2.3
Fe^{2+} sat $2p_{1/2}$	2.8	3.6	2.5	2.8	2.5	1.7	2.0	2.6	1.2
Fe^{3+} sat $2p_{1/2}$	3.4	4.3	2.0	5.0	6.7	3.0	1.0	3.1	1.8

[a] oct, octahedral; sat, satellite

[b] obtained via curve-fitting in this work
